# Primary health care teams put to the test a cross-sectional study from Austria within the QUALICOPC project

**DOI:** 10.1186/s12875-015-0384-9

**Published:** 2015-11-16

**Authors:** Kathryn Hoffmann, Aaron George, Thomas E. Dorner, Katharina Süß, Willemijn L. A. Schäfer, Manfred Maier

**Affiliations:** Department of General Practice, Center for Public Health, Medical University of Vienna, Kinderspitalgasse 15/1st floor, 1090 Vienna, Austria; Department of Community and Family Medicine, Duke Medical Center, Durham, NC USA; Institute for Social Medicine, Center for Public Health, Medical University of Vienna, Vienna, Austria; NIVEL, The Netherlands Institute for Health Services Research, Utrecht, The Netherlands

**Keywords:** Primary Care, Primary health care team, Psychosocial care, Preventive care, Work satisfaction, Austria

## Abstract

**Background:**

Multidisciplinary Primary Health Care Teams (PHCT) provide a comprehensive approach to address the social and health needs of communities. It was the aim of this analysis to assess the number of PHCT in Austria, a country with a weak PHC system, and to compare preventive activities, psychosocial care, and work satisfaction between GPs who work and those who do not work in PHCT.

**Method:**

Within the QUALICOPC study, data collection was performed between November 2011 and May 2012, utilizing a standardized questionnaire for GPs. A stratified sample of GPs from across Austria was invited. Statistical analyses included descriptive statistics and tests.

**Results:**

Data from 171 GPs questionnaires were used for this analysis. Of these, 61.1 % (*n* = 113) had a mono-disciplinary office, 26.3 % (*n* = 45) worked in an office consisting of GP, receptionist and one additional primary care profession, and 7.6 % (*n* = 13) worked in a larger PHCT. GPs that worked in larger PHCT were younger and more involved in psychosocial and preventive care. No differences were found with regard to work satisfaction or workload.

**Conclusions:**

This study gives insight into the structures of PHC in Austria. The results indicate a low number of PHCT; however, the overall return rate in our sample was low with more male GPs, more GPs from urban areas and more GPs working in offices together with other physicians than the national average. Younger GPs demonstrate a greater tendency to implement this primary care practice model in their practices, which seems to be associated with an emphasis in psychosocial and preventive care. If Austria is to increase the number of PHC teams, the country should embrace the work of young GPs and should offer relevant support for PHCT. Future developments could be guided by considering effective models of good practice and governmental support as in other countries.

**Electronic supplementary material:**

The online version of this article (doi:10.1186/s12875-015-0384-9) contains supplementary material, which is available to authorized users.

## Background

Primary Health Care Teams (PHCT) aim to provide a comprehensive approach to address community needs through assessment of, and response to, local social and health deficits [1–6]. Historically, the PHCT was a systemic approach designed to provide long-term care for patients and to be inclusive of the bio-psycho-social context of living. Prior to the development of the PHCT model, the declaration of Alma-Ata in 1978 recognized the essential role that social determinants of health play in disease development and related prevention. Alongside this, it was generally agreed that GPs alone could not handle and address all of these factors simultaneously [1, 3, 7–11]. Ideally, the composition and size of the team would be need-based based on the composition of the practice population or community where the practice is located [3]. In this sense, a PHCT is defined conceptually as team consisting of different primary care professionals who work together, responding to and reflecting the health promotion, disease prevention, curation, and rehabilitation needs of a defined group of persons [3, 7]. A number of studies have demonstrated the benefits of PHCT, which include improved work satisfaction and quality of life for all team members, as well as increased patient satisfaction [2–6, 10–12]. These improvements were identified in those teams that modeled the recommendations of Xyrichits and Lowton in relating team premises, team size and composition in accordance with the health needs of the related population, organizational support, team meetings, clear goals and objectives, and audit [13]. Moreover, the trans-disciplinary approach of PHCT has the potential to provide both more psychosocial and preventive services and better community-oriented, patient-centered activities [2, 14–16]. In addition, patients that visit a PHCT stand to benefit from improved health outcomes, particularly the elderly and patients with mental diseases, chronic conditions and multiple co-morbidities [14–19].

Recognizing these advantages and outcomes, it is becoming increasingly evident that the PHCT is an effective and ideal model for delivering comprehensive primary health care services [8, 9]. Along this line, an independent expert panel named EXPH was convened by the European Commission in 2014, strongly arguing for a primary-care system that is provided by a team of professionals [3]. Further to this, a recent Lancet editorial pointed out that “*[….] The time of the lone general practitioner is over and outdated* [1]*.”*

However, the majority of the studies on the benefits of PHCT cited above have been conducted in countries with recognized strong primary health care systems and long-standing traditions of PHCT. In these study settings, specific education and training of health professionals in the primary care sector is common and well-regarded. Further, many of these successful models for PHCT function through structures such as Primary Health Care Centers or Medical Homes which are maintained and financed through support by government or federal states. In contrast, nearly no data on PHCT are available from countries with a relatively weak primary care system, as classified via the Primary Healthcare Activity Monitor for Europe by Kringos et al. [20, 21], such as Austria.

### The Austrian context

While it is legally possible to establish a PHCT (GPs and other primary care professions) or a group-practice (two or more physicians (GPs and/or specialists) working together) under one roof in Austria, there exist many structural, financial and administrative barriers compared to a traditional solo-practice. For example, all physicians in Austria working in the ambulatory sector are required to be self-employed; therefore, it is legally not possible for a physician to employ another colleague [22]. If a GP wants to establish a PHCT model, it would necessitate payment of salaries for receptionists, nurses, lab assistants or other health professionals from his or her income. Meanwhile, GP income is heavily dependent upon the number of GP-patient contacts in a system without list- and gatekeeping system [22, 23]. In other words, there is no specific remuneration system for PHCT. Moreover, in Austria there is no special education or training yet available for nurses working in primary health care practices. This presents a significant challenge to promoting and expanding PHCT, as there are no personnel, aside from GPs, with the scope of practice rights to triage, diagnose, prescribe medications or lead community-outreach services [24]. This lack in specific training for nurses is similar for other health professionals, such as health secretaries [25].

### Aims and objectives

Against this background, the present study aimed to assess the number of PHC teams in the Austrian context and the demographic variables of GPs working in PHCT. Further goals included the comparison of preventive activities and psychosocial care provided, and of the work satisfaction between the groups of GPs working in PHC teams with those who do not.

## Methods

### Design

This cross-sectional study was conducted within the framework of the European QUALICOPC study [26–28]. The data collection took place between November, 2011 and May, 2012. The study analysis was designed in accordance with the STROBE statement for cross-sectional studies (http://www.strobe-statement.org/index.php?id=available-checklists).

### Recruitment of the sample

The target for Austria given by the QUALICOPC design was to recruit 180 GPs from each of the nine federal states, with representation of sexes, various age groups, and both GPs with and without contracts with public social health insurance companies. The inclusion criterion was that the GP had to have a medical office in Austria. Moreover, only one GP per office was included.

Due to the non-availability of an email or telephone list of all practicing GPs in Austria (*n* = 6527), GPs that were members of the Austrian Society for General Practitioners, as well as GPs that had a valid email address on the websites of the federal societies of the Austrian Chamber of Physicians, were invited via email to participate in the QUALICOPC study. Following, 1828 GPs were invited electronically. Up to three reminders were sent to these GPs with more reminders to geographical areas with fewer replies. Overall, 196 GPs primarily agreed to participate (return rate 10.7 %) but three did not meet the inclusion criteria and nine, ultimately, could not participate due to time constrains. We reviewed the data for the remaining 184 GPs carefully in terms of their similarity with the Austrian GP sample for the year 2011 (Additional file 1) and point out potential limitations in the discussion section since our sample included more male GPs, more GPs from urban areas and more GPs working in group-practices than the national average. The data were provided by the Austrian Chamber of Physicians in terms of sex, age, distribution between the nine federal states and regarding their solo- or group-practice (practice together with another physician) status [29].

### Definition of PHCT status

The EXPH suggests that a typical primary care team includes, amongst others, GPs, nurses, pharmacists, optometrists, dentists, dieticians, midwives, physiotherapists, psychotherapists, occupational therapists, and social workers [3].

For the purposes of this study, we differentiated the PHCT status into the following groups:

- Mono-disciplinary office (GP (+ receptionist)): Although it is possible that receptionists can pass courses regarding basic health care skills and, therefore, may also function as a kind of basic health assistants under the supervision of the GP [25], we considered these offices not as PHCT.
- Small PHCT (GP + receptionist + at least one other health professional such as a nurse, physiotherapist, or lab assistant etc.)
- Larger PHCT (GP + receptionist + at least two other health professionals)

The differentiation between small PHCT and larger PHCT was made on the basis that the vast majority of care needs of the public according to the literature are ideally maintained by larger groups through community-oriented, appropriate responses [1, 3, 9].

### Questionnaire

The development of the questionnaire was a four-phase approach, including a pilot survey, and is further described by Schäfer et al: the four phase approach consisted of a search for existing validated questionnaires, the classification and selection of relevant questions, shortening of the questionnaire in three consensus rounds, and the pilot survey [27]. After this process, the questionnaire was first translated into the respective languages by the country coordinators and, then, back into English to avoid possible errors within the translation process.

#### Dependent variable

The questionnaire for GPs contained 60 questions. The dependent variable “PHCT status” was surveyed utilizing the question: “Which of the following disciplines are working in your practice/center?”, with the answer categories: receptionist/medical secretary/health assistant, practice nurse, community/home care nurse, psychiatric nurse, nurse practitioner, assistant for laboratory work, manager of the center or practice (not a physician), midwife, physiotherapist, dentists, pharmacists and social worker. The answers were clustered into the three GP office workforce status groups described above. The answer categories of practice nurse, community nurse, and nurse practitioner were all considered together as “nurse” working in a GP office.

#### Independent variables

The following questions were surveyed to address preventive activities: “Does your practice nurse or assistant independently provide: immunization/health promotion/routine checks of chronically ill patients/minor procedures like wound treatment or ear syringing?” For the calculation of this question we considered only mono-disciplinary offices with a secretary which might be able to function as a kind of health assistant as described above (*n* = 69) and excluded the GPs that worked completely alone (*n* = 44). A second question regarding preventive activities was “During the past 12 months, have you offered (a) special session(s) or clinics for the following groups? Diabetic patients/ hypertensive patients/elderly” with the answer categories “yes” or “no”. All GPs were taken into account for calculation of these questions.

To address psychosocial care the following question was surveyed: “In case of the following health problem, to what extent will patients in your practice population (people who normally apply to you for primary medical care) contact you as first health care provider? Physically abused child aged 13/ couple with relationship problems/ man aged 32 with sexual problems/ man aged 52 with psycho-social problems” with the answer categories “almost always”, “usually”, “occasionally”, or “seldom/never”.

Work satisfaction was assessed with the following questions: “I feel that some parts of my work do not really make sense”, “My work still interests me as much as it ever did”, “My work is overloaded with unnecessary administrative detail”, “I have too much stress in my current job”, and “In my work there is a good balance between effort and reward” with the answer options “strongly agree”, “agree”, “disagree”, “strongly disagree” (the answer options were later dichotomized into “agreed” and “disagreed”).

As demographic variables, age and sex were surveyed, as well as the place of the GP office. The answer options were “big city”, “suburbs”, “small town” and “rural area” (these categories were dichotomized into urban (big cities, suburbs, small town) and rural (intermediate and rural) areas). Finally, GP office related variables were addressed as follows: “Are you self-employed with contract(s) with social health insurance companies?”/“Self-employed without contract(s)?”, “How many hours per week do you work as a GP (excluding additional jobs and on-call duties)?”,

“In the past 3 working months (excl. holidays etc.) how often did you have on-call duties during evenings, nights and weekends?”, “What is the (estimated) size of your practice population?” and “Do you work alone or in shared accommodation with one or more GPs and/or medical specialists?”.

### Data analysis

Descriptive analyses were conducted by using cross-tabs. For subgroup analyses between the PHCT status groups, the statistical tests applied was the Chi-Square Independency test, including Fishers’ Exact Test for small sample size analyses or the one-way ANOVA. For each of the psychosocial care questions a single score was built: the answer “almost always” got the most points (four), the answer “seldom/never” the least points (one), all others in between accordingly.

If an independency in the Chi-Square test could not be proven, the z-test, including the Bonferroni method for multiple testing, was applied to learn which sub-groups exactly were dependent. The significance level for all calculations was *p* < 0.05, the confidence interval 95 %.

SPSS Statistics 21.0 was used for all analyses.

### Ethical considerations

The QUALICOPC study and this analysis for Austria were approved by the Ethics Committee of the Medical University of Vienna (EC #808/2011). All participants had to sign a written informed consent form before their participation in the study.

## Results

Overall, data from 171 questionnaires out of 184 which were returned could be analyzed. 13 questionnaires had to be excluded because the PHCT status questions were not answered. The mean age of the participating GPs was 54.3 years (SD 7.3, range 34-72 years), 29.3 % were female (3.3 % gave no answer), 55.4 % lived in urban, and 41.3 % in rural areas (3.3 % marked no answer). In relation to the practice location, 33.2 % had their practice in the federal state Vienna, 18.5 % in Lower Austria, 16.8 % in Styria, 10.9 % in Upper Austria, 5.4 % in Burgenland, 4.9 % in Salzburg and Tyrol each, and 2.7 % in Carinthia and Vorarlberg each. Regarding the group-practice working status, 85.9 % were the only physician in their offices, 13.0 % (*n* = 22) worked in group-practices (1.1 % gave no answer), 12 GPs shared the practice/office with other GP(s), eight with other specialist(s), and four with both (see also additional file 1).

### Low occurrence of PHCT

Of the GPs surveyed in this study:

- 61.1 % (*n* = 113) had a mono-disciplinary office (*n* = 44 GP only, *n* = 69 GP + receptionist)
- 26.3 % (*n* = 45) worked in a small PHCT (all 45 had receptionist(s), in addition, 31 GPs had a nurse working in their office, five GPs worked together with an assistant for laboratory work, eight GPs with a physiotherapist, and one GP worked together with a dentist)
- 7.6 % (*n* = 13) worked in a larger PHCT (all had receptionist(s) and a nurse, additionally, 4 GPs worked together with a laboratory assistant, one GP with a midwife, ten GPs with a physiotherapist, three with a social worker, and one GP additionally had a manager)

Table 1 shows the distribution of the demographic variables for the GPs related to a certain PHCT status and presents statistical significant differences. We identified significant associations between younger age, rural location of the practice, working in group practices together with other physicians and working in PHCT.

**Table 1:** Distribution of the demographic variables for the GPs related to a certain PHCT status

		Mono-disciplinary	Small PHCT	PHCT	*p*-value (Fishers Exact Test)
Variable	Subgroup	% (*n*)	% (*n*)	% (*n*)	
Age	34-44 years	33.3 (6)_a_	50.0 (9)_a_	16.7 (3)_a_	0.026
	45-54 years	72.2 (39)_b_	25.9 (14)_a_	1.9 (1)_a_	
	55-64 years	69.4 (59)_b_	21.2 (18)_a_	9.4 (8)_a_	
	65-72 years	75.0 (6)_a_	25.0 (2)_b_	(0) _a,b_	
Sex	Female	77.1 (37)_a_	18.8 (9)_a_	4.2 (2)_a_	0.211
	Male	62.4 (73)_a_	29.1 (34)_a_	8.5 (10)_a_	
Location of office	Urban	73.9 (68)_a_	18.5 (17)_a_	7.6 (7)_a_	0.044
	Rural	57.5 (42)_b_	35.6 (26)_b_	6.8 (5)_a_	
Group practice	yes	36.4 (8)_a_	45.5 (10)_a_	18.2 (4)_a_	0.004
	no	71.4 (105)_b_	22.4 (33)_b_	6.1 (9)_b_	

a, b The subscript letters following the percentages and total numbers (a, b,) represent a subset of the variable category that is not significantly different at a significance level of *p* < 0.05 if it is the same subscript letter for the same PHCT status

*p*-value significant at the level of *p* < 0.05

Regarding possible confounders, there were no differences observed in PHCT status groups between GPs with (97.2 % vs 97.6 % vs 83.3 %; p > 0.05) and without established contracts (2.8 % vs 2.3 % vs 16.7 %; p > 0.05) with public social health insurance companies. Moreover, the PHCT groups did not differ in the estimated size of the practice population (3,351 SD 2,762 vs 4,377 SD 4,226 vs 2,591 SD 1,592; *p* = 0.124) or the mean consultation time in minutes (10.0 SD 7.1 vs 9.5 SD 5.9 vs 10.6 SD 9.8; *p* = 0.873). Further, no difference was found in relation to the number of working hours (43.8 SD 12.4 vs 47.2 SD 9.5 vs 41.0 SD 12.1; *p* = 0.182) within a week (during office hours), the number of night-shifts during the last three months (6.1 SD 9.8 vs 7.3 SD 10.6 vs 13.8 SD 25.4; *p* = 0.065), or the number of weekend-shifts during the last three months (3.1 SD 5.2 vs 4.2 SD 4.2 vs 2.3 SD 1.6; *p* = 0.340).

### Preventive activities and psychosocial care more frequent in PHCT

From the mono-disciplinary practices and the group of the small PHCT, 38.9 % (*n* = 44) and 8.9 % (*n* = 4) respectively, indicated that they did not have an assistant or a nurse who independently provided any of the preventive tasks. Table 2 shows the differences between the PHCT status groups (mono-disciplinary offices with secretaries, small PHCT and larger PHCT) in relation to the tasks independently provided by their assistant or nurse. This suggests that assistants or nurses working in larger PHCT more frequently perform those tasks independently.

**Table 2:** Differences between the PHCT status groups in relation to the question, “Does your practice nurse or assistant independently provide?”

		Mono-disciplinary (*n* = 69)	Small PHCT (*n* = 41)	PHCT (*n* = 11)	*p*-value (Fishers Exact Test)
Variable		% (n)	% (n)	% (n)	
Immunization	yes	7.1 (5)_a_	46.3 (19)_b_	63.6 (7)_b_	<0.001
Health promotion (giving lifestyle advise)	yes	18.6 (13)_a_	41.5 (17)_a_	81.8 (9)_b_	<0.001
Routine checks of chronically ill patients	yes	40.0 (28)_a_	53.7 (22)_a_	90.9 (10)_b_	0.004
Minor procedures	yes	22.9 (16)_a_	39.0 (41)_a_	72.7 (11)_b_	0.003

a, b The subscript letters following the percentages and total numbers (a, b) represent a subset of the variable category that is not significantly different at a significance level of *p* < 0.05 if it is the same subscript for the same variable

*p*-value significant at the level of *p* < 0.05

There was a significant difference observed concerning the offering of special services for the elderly (4.4 % vs 17.8 % vs 30.8 %; *p* = 0.005), showing that larger PHCT provide significantly more special sessions for the elderly. However, no difference could be found between the three different PHCT status groups that indicated they included special sessions or clinics for diabetic patients (46.0 % vs 57.8 % vs 46.2 %; *p* = 0.626) or hypertensive patients (14.2 % vs 8.9 % vs 23.1 %; *p* = 0.487).

Moreover, GPs working in larger PHCT models indicated a higher likelihood for patients to contact them as a first health provider, and this was found to be statistically significant in two of the presented cases (Table 3). Specifically, these larger PHCT more often marked “almost always” in response to the question of whether they would be the first health care provider for a physically abused child aged 13, a couple with relationship problems, and a man aged 32 with sexual problems.

**Table 3:** Differences between the PHCT status groups in relation to the psychosocial health care score

	Mono-disciplinary	Small PHCT	PHCT	*p*-value (ANOVA)
Variable	Mean (SD)	Mean (SD)	Mean (SD)	
Physically abused child (13a)	1.58 (0.79)	1.72 (0.80)	2.23 (1.30)	0.029
Couple with relationship problems	1.95 (0.80)	2.10 (0.75)	2.31 (1.32)	0.312
Woman with psychosocial problems (50a)	2.86 (0.82)	2.81 (0.76)	3.00 (1.00)	0.775
Man with sexual problems (32a)	2.31 (0.67)	2.28 (0.85)	2.85 (0.90)	0.040

*p*-value significant at the level of *p* < 0.05

### Overall low work satisfaction of GPs

Table 4 shows the variables for self-reported work satisfaction in relation to the PHCT status. No statistical significant differences could be found for any of these variables, only a trend for larger PHCT to have more interest in their work and a higher perceived balance between work effort and reward. Additionally, larger PHCT reported to have less unnecessary administrative workload than the other groups (non-significant).

**Table 4:** Differences between the PHCT status groups and work satisfaction variables

		Mono-disciplinary	Small PHCT	PHCT	p-value (Fishers Exact Test)
Variable		% (n)	% (n)	% (n)	
Some parts of my work do not really make sense	Agree	24.8 (28)	22.2 (10)	23.1 (3)	0.957
My work still interests me as much as ever	Agree	88.5 (100)	97.8 (44)	100 (13)	0.124
My work is overloaded with unnecessary administrative detail	Agree	85.0 (96)	84.4 (38)	69.2 (9)	0.366
I have too much stress	Agree	63.7 (72)	68.9 (31)	61.5 (8)	0.811
GP is a well-respected job	Agree	69.0 (78)	72.7 (32)	69.2 (9)	0.927
In my work there is a good balance between effort and reward	Agree	37.5 (42)	29.5 (13)	46.2 (6)	0.464

*p*-value significant at the level of *p* < 0.05

## Discussion

The most important finding of this study reveals for the first time an insight into the structures of PHC in Austria and a low number of PHCT under one roof in Austria. Nearly two-thirds of offices included did not have any supportive health providers in their practice, while a further one-quarter consisted of only three different professions: physician, receptionist, and another staff member. This raises the question as to whether these three profession units are truly reflective of a PHCT even at a small scale. Recent considerations for PHCT composition, as well as the recent definition proposed by the EXPH, recognize these teams as more consistent with substantial trans-disciplinary groups that jointly coordinate care and act accountable for the large majority of the communities’ health needs [3]. It is unlikely that a solo physician with a receptionist and one other team member can adequately be accountable for the full spectrum of social and health needs of a respective community as defined as the duty of primary care services [1, 3, 7–9, 30, 31]. This is particularly important in light of the training situation for certain health professionals in Austria as described in the background section.

Only 7.6 % of offices were identified as having a staff consistent with a larger PHCT (including a GP, receptionist, nurse, physiotherapist, or social worker). This implies that it would be helpful for future Austrian primary health care systems to invest in the development of PHCT, with team compositions that are reflective of the needs of the community. Effective models to emulate would include the Community Health Centers in Belgium [3] or, since the costs of additional personnel can be quite large, structured networks of existing primary care professionals for certain areas like a German project including its shared-savings incentive [32, 33].

GPs indicated that their assistant could independently perform basic preventive tasks, such as health checks for chronically ill patients or vaccinations (Table 2). This seems to be a misinterpretation of the word “independently” as it is not legally allowed for a health secretary or nurse in Austria to engage in diagnostics or medical treatment unless previously instructed by a physician [24]. It is likely that assistants participated in some elements of disease management programs on their own or administered vaccination independently after being authorized by a physician. In such scenarios, GPs may not necessarily have altered the plan, so these GPs might have perceived the work of the assistant as more or less independent. This perception of assistant independence is more prominent in larger PHCT practices. However, it appears that GPs working in larger PHCT would benefit from additional health care professionals to conduct special tasks without instruction. Meanwhile, one recent U.S. study found out those organizational cultures that emphasize collegiality and quality but not autonomy were related to quality evaluation and improvement [34].

One clear outcome of our study is the identification that young physicians are much more likely to work in teams structured like PHCT. Additionally, rural location of practice, as well as the ability to work in a group practice together with other physicians, could additionally be identified with working in a PHCT (Table 1). The overall higher practice population in rural areas with patients with greater diversity of physical and psychosocial needs may make care by a single provider unmanageable. This finding is reflected by previous studies that showed the need for and effectiveness of primary care teams, particularly for those in rural areas [33, 35–37].

There were no differences observed in the working hours, consultation time, and work satisfaction between the PHCT and the solo-GP groups (Table 4). There are some possible explanations for this finding, such as if non-GP team members substitute tasks of the GPs to allow time for more complex and challenging patient care. However, other team members may not substitute those tasks of the GPs, but rather take on extra tasks such as physiotherapy. One previous study showed that primary care teams provide more comprehensive services and generate higher patient satisfaction as a result of these services. Because of this satisfaction, these primary care teams will often see a higher number of patients with complex conditions, particularly with mental health conditions, leading to more challenging and time-consuming work for the team [14]. However, the identification of no differences in work satisfaction variables between the PHCT groups is in contrast to previous studies [2, 4–6, 10, 12, 19, 33]. Perhaps, this finding may be more a consequence of the overall structure of the Austrian health care system with its mixed reimbursement system and fee-for-service payments, than to the specific structure of the practices. It is additionally noteworthy that the stress level of all GPs is elevated, with around 65 % expressing high levels of stress. Further, approximately 80 % of GPs endorsed the feeling that their work is overloaded with unnecessary administrative details. Concurrently, only about 38 % of GPs suggested they felt a good balance between effort and reward in their job (Table 4).

Despite these observed challenges, greater involvement in psychosocial services and services for the elderly were observed with PHCT (Table 3). This finding is supported by several studies which showed that while PHCT see a higher number of persons with mental health conditions and complex chronic conditions, these teams continue to demonstrate better health outcomes [14–18, 32, 33]. One recent US study suggested that team-based primary care may represent the most critical method used to successfully transform primary care services towards meeting the needs of complex and high-risk patients and improving patient, provider, and employee satisfaction [10].

The major strength of this study is that it is the first survey to comparatively assess team-based versus solo GP care in a European country with a relatively weak primary health care system, along with consideration for its composition and impact. Additionally, the sample of GPs studied is roughly similar to the national situation in Austria [29]. The distribution of Austrian GPs in the year 2011 was 39 % female, the mean age was 52.5 years, the distribution between the nine federal states was 21.4 % for Vienna, 19.6 % for Lower Austria, 16.6 % for Upper Austria, 14.7 % for Styria, 7.6 % for Tyrol, 6.7 % for Carinthia, 6.5 % for Salzburg, 3.6 % for Vorarlberg, and 3.3 % for Burgenland. Group-practices comprised only 8 % of those GP practices in Austria (additional file 1). It must be acknowledged that there are differences in our sample with regard to the distribution of sex, to GPs practicing in Vienna and with regard to GPs working in group practices. Statistical differences are shown in additional file 1. It can be concluded that the overrepresentation of males and group practice owners in our sample could have led to an overestimation of the number of PHCT, as group practice is one factor that was found to be associated with working in larger PHCTs. Meanwhile, the overrepresentation of GPs with offices in Vienna, which is at the same time the capital and an urban region, could have led to an underestimation of PHCT which are associated with GPs working in rural areas.

Another limitation of this study is the sample size, particularly the size of the PHCT subgroup.. This was a limiting factor in the calculation of statistically significant results between the subgroups. The major limitation, however, might be the potential selection bias due to the low response rate and the voluntary participation of GPs. Though, low response rates are a well-known problem and a particular challenge in GP research [38–40]. In 2014, Parkinson and colleagues demonstrated this difficulty in recruiting GPs for surveys, showing that a response rate of even 14.5 % required great effort [41]. Further, Rumball-Smith and colleagues showed a response rate of 12.2 % for the QUALICOPC sample in New Zealand [15]. This limitation was also discussed in the comparative analysis of the QUALICOPC project in the Bulletin of the World Health Organization [28]. Additionally, the low response rate is related to the current status and low interest in PHC research, the organization of the GP workforce in Austria and the fact that GPs are relegated to use their own free time for all research activities. This could have led to a selection of only highly motivated, well-organized GPs with interest to complete the questionnaire, which could produce an overestimation of GPs working in PHCT and of the work satisfaction of the sample studied. Conversely, the selection process could have led to a bias with a higher response from those GPs that are unhappy with their daily work and want to change the situation. Overall, this bias affects the generalizability of the findings. There are insufficient supporting data to investigate the representativeness of the samples at this time, so we cannot specify the impact of potential response bias.

Another limitation is the fact that this analysis was conducted entirely within the context of the QUALICOPC study and its questionnaire. Following, for example, for the variable “job satisfaction” we used six questions out of the QUALICOPC questionnaire, and did not employ any further validated questionnaires. This could have led to some uncertainty in the estimation of job satisfied/dissatisfied GPs. Furthermore, the term “independently” as used in the questionnaire and in defining a practice has generated obvious misinterpretations and provides room for speculation that the GPs could have misinterpreted other terms within the questionnaire. An additional qualitative study on the understanding of GPs of this and other certain terms would be beneficial to aid in more precise interpretation of these results. Moreover, we built a score for each of the psychosocial questions that was not validated. However, it was our intent for this score to provide an initial impression of the current situation in Austria. Additionally, this is a cross-sectional study which does not allow conclusions about casual relationships.

## Conclusion

In summary, the number of PHCT in Austria seems to be rather low. Though, young physicians did demonstrate a higher likelihood to work in teams. Both, working in rural practices as well as working in a shared group practice with other physicians were factors associated with an increased likelihood to work in a PHCT. Increased provisions of psychosocial services and services for elderly were observed in larger PHCT groups, as compared to all other practice types.

If Austria is to increase the number of PHC teams, the country should embrace and support the initiatives of young GPs to work in groups and PHCT. There are currently few incentives and only limited support to advance team-based care in Austria. Further, new medical training models should be developed to provide the diverse competencies needed in a full PHCT and to enable the members to successfully work in trans-disciplinary teams responding to the health needs of the population. We believe that our findings may be relevant for other countries which try to develop and strengthen primary health care systems, particularly those with a substantial number of primary care providers who are self-employed and working in solo-practices.

## Additional file

Additional file 1:
**Comparison of demographics of the GPs in the sample with the Austrian GP population.** (DOCX 16 kb)

## Abbreviations

ANOVA: Analysis of variance
EXPH: Expert Panel on Effective Way of Investing in Health
GP: General Practitioner
PHCT: Primary Health Care team(s)
QUALICOPC: Quality and Costs in Primary Care
