# Supplementary material for: Primary health care teams put to the test a cross-sectional study from Austria within the QUALICOPC project
Source: BMC Fam Pract. 2015 Nov 16;16:168. doi: 10.1186/s12875-015-0384-9 (PMC4647311; doi:10.1186/s12875-015-0384-9)
Supplement: Additional file 1: — Comparison of demographics of the GPs in the sample with the Austrian GP population. (DOCX 16 kb) [file 12875_2015_384_MOESM1_ESM.docx]

**Additional file 1.** Comparison of demographics of the GPs in the sample with the Austrian GP population

| Varable | Sub-variable | GP sample of the study (n=171) | Austrian GP population (n=6527) | Statistically significant differences* |
| --- | --- | --- | --- | --- |
|  |  | Mean, CI 95% (SD) | Mean, CI 95% (SD) |  |
| Age |  | 54.3, 52.2-55.4 (7.3) | 52.2, 51.9-52.5 (6.2) | no |
|  |  | %, CI 95% (n) | %, CI 95% (n) |  |
| Sex | Female | 29.3, 24.4-36.2 (50) | 39.0, 36.8-41.3 (2546) | yes |
|  | Male | 67.4, 63.8-75.6 (115) | 61.0, 58.7-63.2 (3981) | yes |
|  | No answer | 3.3 (6) | - |  |
| Federal state | Vienna | 33.2, 25.6-40.9 (57) | 21.4, 19.6-23.3 (1397) | yes |
|  | Lower Austria | 18.5 12.9-25.5 (32) | 19.6, 17.9-21.3 (1279) | no |
|  | Upper Austria | 10.9, 6.3-16.7 (19) | 16.6, 14.9-18.3 (1084) | no |
|  | Styria | 16.8, 10.3-22.4 (28) | 14.7, 13.2-16.4 (960) | no |
|  | Tyrol | 4.9, 1.7-8.5 (8) | 7.6, 6.4-8.8 (496) | no |
|  | Carinthia | 2.7, 0.6-5.7 (5) | 6.7, 5.6-8.0 (437) | no |
|  | Salzburg | 4.9, 2.2-7.9 (8) | 6.5, 5.4-7.7 (424) | no |
|  | Vorarlberg | 2.7, 0.8-6.0 (5) | 3.6, 2.8-4.5 (235) | no |
|  | Burgenland | 5.4, 1.4-7.6 (9) | 3.3, 2.4-4.1 (215) | no |
| Office organization | Solo-office | 85.9, 80.8-91.2 (147) | 92.0, 90.8-93.2 (6005) | yes |
|  | Group practice | 13.0, 8.4-19.2 (22) | 8.0, 6.8-9.2 (522) | yes |
|  | No answer | 1.1 (2) | - |  |

* Significant at a significance level of p<0.05
